# Increased serum brain-derived neurotrophic factor in male schizophrenic patients with metabolic syndrome

**DOI:** 10.1097/MD.0000000000007089

**Published:** 2017-06-02

**Authors:** Chin-Chuen Lin, Yi-Yung Hung, Meng-Chang Tsai, Tiao-Lai Huang

**Affiliations:** Department of Psychiatry, Kaohsiung Chang Gung Memorial Hospital and Chang Gung University College of Medicine, Kaohsiung, Taiwan.

**Keywords:** brain-derived neurotrophic factor, gender difference, metabolic syndrome, schizophrenia

## Abstract

Increased prevalence of metabolic syndrome was found in patients with schizophrenia. Brain-derived neurotrophic factor (BDNF) was involved in energy metabolism and the pathophysiology of schizophrenia, but differently in males and females. We aimed to investigate the serum BDNF levels in patients with schizophrenia with and without metabolic syndrome.

Patients with schizophrenia were recruited. Their demographic data were collected. Metabolic profiles and serum BDNF levels were measured. Clinical symptoms were evaluated with Positive and Negative Syndrome Scale. Metabolic syndrome was determined with the criteria provided by Ministry of Health and Welfare of Taiwan. Framingham Risk Score (FRS) for estimate of 10-year risk for coronary heart disease was provided by National Institutes of Health.

Of the 81 participants, 40.7% had metabolic syndrome. Those with metabolic syndrome had higher FRS. Using analysis of covariance adjusted for age and body mass index, male patients with schizophrenia with metabolic syndrome had higher serum BDNF levels than those without (4.6 ± 4.7 vs 3.3 ± 3.8 ng/mL, *P* = .022). No statistical difference was found between female patients with and without metabolic syndrome.

Significant differences of serum BDNF levels were found between male patients with schizophrenia with and without metabolic syndrome, but not in females. This finding suggested the gender difference behind the mechanism of BDNF in metabolic syndrome in schizophrenia.

## 1 Introduction

Brain-derived neurotrophic factor (BDNF), a key member of the family of neurotrophic factors that play important roles in the growth, survival, differentiation, and repair of neurons, is an important marker of neurodevelopmental processes.^[1]^ BDNF has also been associated with neuropsychiatric disorders, such as schizophrenia and mood disorder.^[2]^ Earlier studies showed BDNF can cross the blood-brain barrier in both directions.^[3,4]^ In animal studies, blood BDNF levels had been shown to correlate positively with cortical BDNF levels.^[3,5]^ In patients with schizophrenia, lower levels of serum BDNF had been reported.^[6]^ BDNF was also involved in energy metabolism, though the findings were not always consistent.^[7–10]^ Furthermore, differences of BDNF levels had been observed between male and female patients with schizophrenia.^[11]^

Patients with schizophrenia were also known to have higher mortality and shorter lifespan.^[12,13]^ Cardiovascular disease might be a major contributing factor to the increased mortality of patients with schizophrenia.^[14,15]^ The diagnosis of metabolic syndrome could be used to identify individuals at risk of cardiovascular disease.^[16]^ The prevalence rates of metabolic syndrome in patients with schizophrenia were about 35% in males and 50% in females, far greater than general population.^[17]^ Many factors, such as antipsychotics, lifestyle, socio-economic status, and genetics, would contribute to the aforementioned phenomenon.^[18]^ In addition to metabolic syndrome, Framingham Risk Score (FRS) is also a powerful tool to detect individuals at risk of cardiovascular disease.^[19]^

In this study, we aimed to investigate the relationship between serum BDNF levels and schizophrenia considering metabolic profiles and gender differences in patients with schizophrenia.

## 2 Methods

### 2.1 Subjects and study design

From July 2015 to June 2016, patients diagnosed with schizophrenia were recruited at the Chang Gung Memorial Hospital. Schizophrenia was diagnosed by a psychiatrist according to the criteria of the *Diagnostic and Statistical Manual of Mental Disorders, Fourth Edition* (DSM-IV).^[20]^ Clinical symptoms were evaluated with Positive and Negative Syndrome Scale (PANSS). Demographic data, blood pressure (BP), waist circumference, prescription of antihypertensive medications, and smoking status were collected. Written informed consent was provided by all participants after the content and context of the study was fully explained. The institutional review board (IRB) of Chang Gung Memorial Hospital approved the study design (IRB 104-2788A3).

A total of 81 patients with schizophrenia were recruited, including 35 males and 46 females. Their ages range from 22 to 77, with the mean of 46.2 ± 10.9. Illness duration range from 1 to 47.2 years, with the mean of 19.5 ± 9.2 years. PANSS scores ranged from 32 to 185, with the mean of 74.8 ± 31.1.

Fourteen (17.3%) patient met 0 criteria of metabolic syndrome; 14 (17.3%) met 1 criterion; 20 (24.7%) met 2 criteria; 16 (19.8%) met 3 criteria; 14 (17.3%) met 4 criteria; and 3 (3.7%) met all criteria. Overall, 33 (40.7%) patients had metabolic syndrome. Among them, 13 of 35 (37.1%) male and 20 of 46 (43.5%) female patients had metabolic syndrome. The male and female distribution of metabolic syndrome is not statistically significant.

In terms of Total Framingham Point Scores, 26 (37%) patients have a score greater or equal to 10. In terms of estimate of 10-year risk of coronary heart disease, 15 (19%) patients have risks greater or equal to 10%. Three patients have risks as high as 25%.

### 2.2 Laboratory data

Venous blood was drawn from each participant in the morning following a 6-hour fast. Levels of fasting blood glucose, triglyceride (TG), high-density lipoprotein cholesterol (HDL-C), and total cholesterol were measured in the hospital laboratory. Serum BDNF protein levels were measured using a commercially available enzyme-linked-immunosorbent serologic assay kit of the sandwich type (BDNF Emax Immunoassay System; Promega, Madison, WI, USA). Each system contained anti-BDNF mAb, Block&Sample 5× buffer, BDNF standard, antihuman BDNF pAb, anti-IgY HRP, TMB solution, peroxidase substrate, and protocol. All samples were assayed or duplicated by the same senior laboratory assistant.

### 2.3 Criteria of metabolic syndrome

Health Promotion Administration of Ministry of Health and Welfare of Taiwan used the following criteria of metabolic syndrome in 2004:

(1) Waist circumference ≥90 cm in males or ≥80 cm in females, or body mass index (BMI) ≥kg/m^2^;
(2) Systolic BP ≥130 mmHg or diastolic BP ≥85 mmHg;
(3) Fasting blood glucose ≥110 mg/dL;
(4) Triglyceride ≥150 mg/dL;
(5) HDL-C <40 mg/dL in males or <50 mg/dL in females.

Patients were considered to have metabolic syndrome if they met 3 or more of the criteria.

### 2.4 Estimate of 10-Year risk for coronary heart disease Framingham Point Scores

The Framingham scores are calculated using formula of age, total cholesterol, smoking status, HDL-C level, and systolic BP, as provided by National Institutes of Health website (https://www.nhlbi.nih.gov/health-pro/guidelines/current/cholesterol-guidelines/quick-desk-reference-html/10-year-risk-framingham-table).

### 2.5 Statistical analysis

All results are represented as mean ± standard deviation. Chi-square was used to analyze the distribution of metabolic syndrome among male and female samples. Independent *t* test was used to compare the Framingham scores between patients with and without metabolic syndrome, as well as serum BDNF levels between male and female patients. Analysis of covariance (ANCOVA) adjusted for age and BMI was used to compare BDNF levels between different groups. Pearson correlation was used to assess the relationship with the associated parameters. Data analysis was performed using SPSS 19 (Chicago, IL). *P* values of <0.05 were considered statistically significant.

## 3 Results

The metabolic profiles are summarized in Table 1. Sixteen (19.8%) patients smoke. Using independent *t* test, patients with metabolic syndrome have higher Framingham scores (8.67 ± 5.54 vs 5.67 ± 6.11, *P* = 0.027).

**Table 1:**
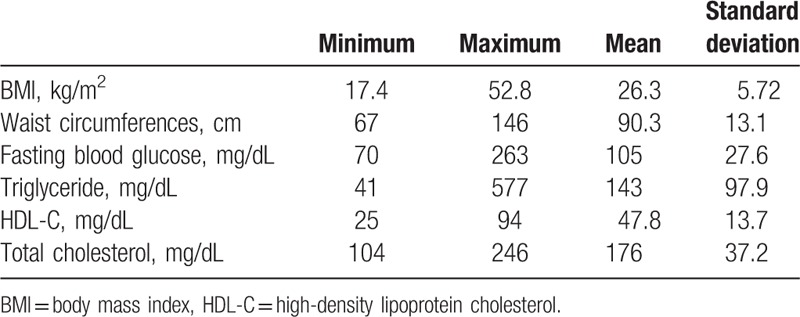
Metabolic profiles.

BDNF levels of patients with metabolic syndrome are 3.7 ± 4.0 ng/mL, whereas BDNF levels of patients without metabolic syndrome are 3.4 ± 4.0 ng/mL. Using ANCOVA adjusted for age and BMI, no statistical significance was found. Using independent *t* test, no statistical significance was found in BDNF levels between male and female patients (3.8 ± 4.1 ng/mL vs 3.3 ± 4.0 ng/mL, *P* = 0.314).

In male samples alone, BDNF levels of patients with metabolic syndrome are 4.6 ± 4.7 ng/mL, whereas BDNF levels of patients without metabolic syndrome are 3.3 ± 3.8 ng/mL. Using ANCOVA adjusted for age and BMI, statistical significance was found between those 2 groups (*P* = 0.022). In female samples, BDNF levels of patients with and without metabolic syndrome are not statistically different (3.0 ± 3.5 vs 3.5 ± 4.3 ng/mL, *P* = 0.274). The results are summarized in Table 2. No significant correlation was found between serum BDNF levels, age, BMI, waist circumference, BP, fasting glucose, TG, HDL-C, total cholesterol, and PANSS score, in either male or female samples (Pearson's r values are −0.179, 0.126, 0.172, 0.261, −0.063, 0.130, −0.142, −0.243, −0.023 for males, and −0.067, 0.009, −0.041, 0.074, −0.129, .−0.046, −0.050, −0.036, −0.240 for females).

**Table 2:**
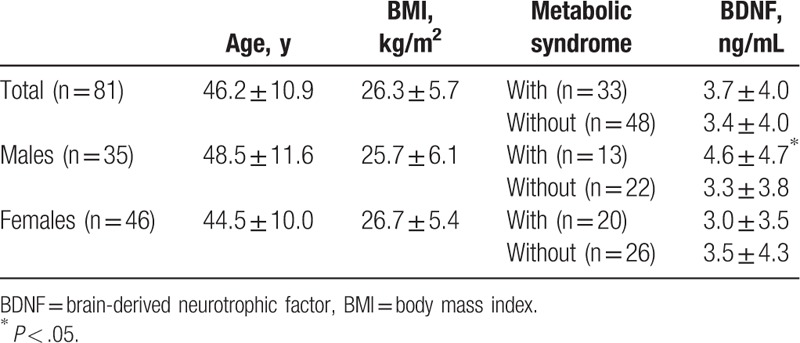
BDNF levels in patients with schizophrenia with and without metabolic syndrome.

## 4 Discussion

The major finding of this study is the gender difference of BDNF levels between ones with and without metabolic syndrome among patients with schizophrenia. In male patients with schizophrenia, ones with metabolic syndrome have significantly higher serum BDNF levels than ones without. This difference is not observed in the female patients with schizophrenia. Gender difference of BDNF levels in patients with schizophrenia have been reported in the past.^[11,21–23]^ Zhang et al^[21]^ reported that no differences in serum BDNF levels were observed between patients with schizophrenia and BMI matched controls; BDNF levels also negatively correlated with BMI gain in female but not in male patients when gender was considered. Xiu et al^[11]^ reported that female patients with schizophrenia had statistically higher serum BDNF levels than male patients, but the gender difference was not found in controls. Ikegame et al^[22]^ found that higher level of methylation at BDNF promoter I was found in patients with schizophrenia compared with controls, and the methylation difference was more prominent in male patients. Kim et al^[23]^ noted that female patients with schizophrenia with Val allele of the BDNF Val66Met polymorphism scored higher in PANSS scale and lower in cognitive tests, a phenomenon not observed in male patients. Although a clearly defined pattern of how BDNF behaves in different genders could not be elucidated yet, those studies agreed on gender being a factor behind the mechanism of BDNF.

The prevalence rate of metabolic syndrome of the study sample was 40.7%, comparative to earlier studies in Taiwan.^[24–26]^ The finding that patients with schizophrenia would have higher 10-year risk of coronary heart disease is also similar to earlier report by Tseng et al.^[26]^ That finding is not surprising given the criteria of metabolic syndrome and Framingham points have overlapping parameters.

There are several limitations to this study. The sample size was relatively small. Many confounding factors were not controlled. The study had a cross-sectional design, which limited the data interpretation.

In this study, significant difference of serum BDNF levels were found between male patients with schizophrenia with and without metabolic syndrome, but not in females. Earlier studies found a gender difference of BDNF levels in patients with schizophrenia, and our finding continued to support this notion. However, no well-defined mechanism could be elucidated yet. Further studies are needed to further clarify the role of gender in BDNF levels in patients with schizophrenia.

## Acknowledgment

This work was supported by a clinical research grant, no. CMRPG8E0401, from Kaohsiung Chang Gung Memorial Hospital in Taiwan.
